# Monitoring Healthy Metabolic Trajectories with Nutritional Metabonomics

**DOI:** 10.3390/nu1010101

**Published:** 2009-09-04

**Authors:** Sebastiano Collino, François-Pierre J. Martin, Sunil Kochhar, Serge Rezzi

**Affiliations:** BioAnalytical Science, Metabonomics & Biomarkers, Nestlé Research Center, PO Box 44, CH-1000 Lausanne 26, Switzerland; Email: Sebastiano.Collino@rdls.nestle.com (S.C.); Francois-Pierre.Martin@rdls.nestle.com (F.P.J.M.); Sunil.Kochhar@rdls.nestle.com (S.K.)

**Keywords:** GC (Gas Chromatography), gut microbiota, MS (Mass Spectrometry), metabolomics, metabonomics, metabotypes, NMR (Nuclear Magnetic Resonance) Spectroscopy, nutrimetabonomics, personalized nutrition, UPLC (Ultra Performance Liquid Chromatography)

## Abstract

Metabonomics is a well established analytical approach for the analysis of physiological regulatory processes *via* the metabolic profiling of biofluids and tissues in living organisms. Its potential is fully exploited in the field of “nutrimetabonomics” that aims at assessing the metabolic effects of active ingredients and foods in individuals. Yet, one of the greatest challenges in nutrition research is to decipher the critical interactions between mammalian organisms and environmental factors, including the gut microbiota. “Nutrimetabonomics” is today foreseen as a powerful approach for future nutritional programs tailored at health maintenance and disease prevention.

## 1. Introduction

Today metabonomics is considered as a well-established system approach to characterize the metabolic phenotype of an individual, which results from a coordinated physiological response to various intrinsic and extrinsic parameters including environment, drugs, diet, lifestyle, genetics and microbiome modulations. Metabonomic analysis refers at the global metabolic profiling of low molecular weight compounds (<1,500 Da) in biofluids (plasma/serum and urine), and tissue extracts or biopsies. Such complex biochemical fingerprints of hundreds, or even thousands, of metabolites reflect the overall metabolic status of an individual, i.e., its metabonome, as the result of highly complex metabolic exchanges within and/or between diverse biological compartments. Since metabolites are the end-products of multiple interactions between biological processes, applications of metabonomics to nutrition sciences provide an exploratory but unique opportunity to depict the molecular mechanisms involved in individual responses to dietary modulations. With the overall aim to provide health maintenance, these scientific challenges are today modulated through the understanding of metabolic disorders and the efficacy of active ingredients.

## 2. Metabonomics Analytical Profiling Techniques

**Figure 1:**
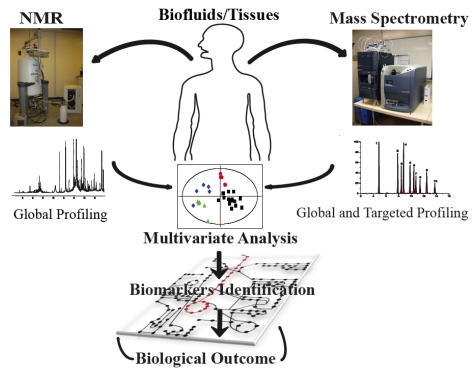
Scheme describing a typical NMR (Bruker 600 MHz) and MS (Waters Acquity Ultra Performance Liquid Chromatograph) based metabonomic analytical platform. NMR offers a holistic profiling of a wide range of metabolites with no *a priori* selection, while MS methods are commonly employed for global and targeted profiling. Both techniques are jointly employed. Multivariate statistical techniques are then used for encoding the complex metabolic profiles, identification of biomarkers to be ultimately used for the elaboration of biological outcome.

The field of metabonomics employs two major analytical techniques based on ^1^H-Nuclear Magnetic Resonance (NMR) spectroscopy, gas or liquid chromatography coupled to mass spectrometry (GC/MS and LC/MS) with lately, the addition of ultrahigh performance liquid chromatography systems coupled to mass spectrometers (UPLC/MS) (Figure 1). High-resolution NMR requires little sample preparation, offers high sample throughput efficacy (10-15 minutes per sample with a conventional detection probe), and high reproducibility. Furthermore, NMR spectroscopy offers the unique prospect, other than elucidating the molecular structures, to holistically and simultaneously profile metabolites with no *a priori* selection. The acquired spectral profile reflects the metabolic imprints of an individual, which adjust in response to a series of patho-physiological stimuli to maintain homeostatic equilibrium. Interestingly, this technique is not only used for the profiling of biological fluids (liquid state NMR), but it is also today commonly employed for the study of metabolic profiling of intact tissue biopsies, using High Resolution Magic Angle Spinning NMR (HR-MAS). Therefore, HR-MAS, presents the unique feature of ensuring the integrity and organizational compartmentation of the biological samples.

However, NMR spectroscopy is inherently less sensitive compared to MS. MS methods are commonly employed for global and targeted profiling, and require well adapted sample preparation [1] with separation of the metabolites components using either gas chromatography (GC) or liquid chromatography (LC) with the more advanced LC (ULPC) being used increasingly. When coupled to chromatographic methods, MS can generate comprehensive metabolic profiles of thousands of signals within a 15-30 min run time thus enlarging the metabolite window for biomarker identification. Multivariate statistical and bioinformatics techniques are ultimately used for data mining the complex metabolic profiles which encapsulate information on genetics, environmental factors, gut microbiota activity, lifestyle, and food habits. This, at the end, sustains the complex process of identifying emerging biomarkers indicative of the individual response to specific physiological factors, and/or nutritional interventions and for the elaboration of biological outcomes (Figure 1).

### 2.1. Nutritional Metabonomics: Deciphering Food-Induced Metabolic Responses

Metabonomics has shown in the past numerous applications in the study of diseases diagnostics, and in the investigation of physiological changes caused by toxic insults [2,3,4,5,6,7,8,9,10,11,12]. Yet, in recent years, metabonomics had been also applied in the area of food research. Here the term “nutrimetabonomics” entirely describes the mutual link among the fields of metabonomic and nutrition research. Recently, to explore how the changes in environmental conditions and lifestyle influence human physiology a large-scale metabonomic study was conducted to investigate metabolic phenotype variation across and within four human populations groups [13]. The investigations performed on 4630 human subjects originating from China, Japan, UK, and USA revealed that the urinary metabolic phenotypes were significantly different for East Asian and Western population samples, with contrasting diets, diet-related major risk factors, and coronary heart disease/stroke rates. Interestingly, it was found that urinary excretion of formate is inversely correlated with blood pressure.

The main goal of nutrimetabonomic studies is therefore to study the effects of selected ingredients and foods in healthy individuals [14], specific dietary metabolic imprints in both human basal metabolism and gut microbiota metabolic activity had been already shown to be closely related, through metabolic profiling of urine, to individual dietary preferences [15]. Additionally, nutrimetabonomics was also used to study the health benefits of caloric restriction (CR) dieting in nonhuman primates [16]. Here, for instance, the authors showed, by applying holistic NMR profiling of blood plasma, marked evidence of reduced aging-depended alteration of energy and lipoprotein metabolism, thus revealing key mechanistic regulatory long terms effects of CR. 

Other works demonstrated the potential of metabolic profiling for surveying the outcome of nutritional interventions for modulating and preventing metabolic deregulations [17,18]. Recently, metabonomics has been applied in combination with measures of blood plasma inflammatory biomarkers and histopathology of gut tissues to elucidate the mechanistic basis and biochemical events behind inflammatory bowel disease (IBD) [19]. By following the gradual development of colitis in a Interleukin 10 knock out mouse model, with selected spontaneous chronic inflammation, holistic metabolic profiling of blood plasma revealed a gradual disruption of energy homeostasis, profound impairment of lipoprotein, phospholipids and polyunsaturated fatty acids metabolism, and an altered glycosylated protein profile. In addition, IL 10 knock out mice displayed higher levels of lactate, pyruvate, citrate, and higher concentration of free amino acids. All together these metabolic changes indicate increased fatty acid oxidation and glycolysis, with higher levels of circulating amino acids reflecting muscle atrophy, increase breakdown of proteins and energy production by interconversion of amino acids. 

Much attention today is also given to the use of probiotic supplements as a means to promote gut health preventing allergies and inflammatory incidences. The effects of a therapeutic intervention with probiotics on normalizing the metabolic disorders acquired in post-infective irritable bowel syndrome were monitored using both systemic and tissue-specific metabolic profiling [20]. These investigations highlighted metabolic discrepancies in relation with muscular hyper-contractility and hypertrophy, and gut microbial disturbance, which were modulated by the probiotic intervention. 

Yet, while many applications in the very next future can be easily envisioned, with personalized nutrition programs as the main objective, nutrimetabonomics remains today an extremely complex science to decipher. Indeed, food-induced metabolic changes are not only the end results of many complex interactions among many active molecules, but also vary extremely among individuals as differences in endogenous factors such as age, environment, genetics, lifestyle, and gut microbiota strongly influence individual responses. For this reason, to maximize the control over these variables and to exclude fictitious interpretations, when analyzing the effects of specific nutritional intervention programs, it is imperative to employ appropriate experimental designs. This is limited most of the time to rigorous inclusion/exclusion criteria and accurate collection of individual dietary and feeding habits.

### 2.2. Nutrimetabonomics: A Specific Tool to Decipher Gut Microbiota Contribution to the Host Metabolic Homeostasis Processes

**Figure 2:**
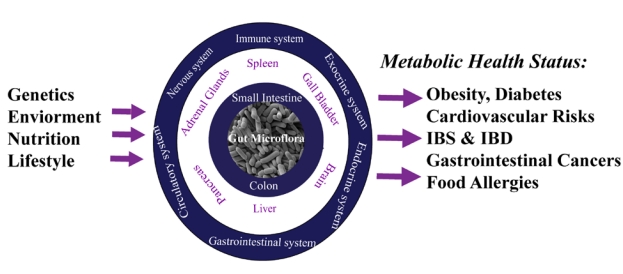
Scheme describing the symbiotic relationship among the host and their gut bacteria, with the gut microbiota exerting a deep control over multiple host cell metabolic regulatory functions.

The contribution of the gut microbiota on the mammalian metabolism had been recently deeply investigated [21,22,23,24,25]. Adult humans carry thousands of gut microbial symbiotic organisms that are in close correlation with the metabolism and immune systems of the hosts [26]. It is now evident that, not only there is a strong relationship among humans and their gut bacteria, but it is now apparent that the gut microbiota exerts a deep control over multiple host cell metabolic pathways [27,28,29]. Recent findings highlighted the gut microbiota as a key determinant in the etiology of many disease, including insulin resistance [30], obesity [31,32], food allergies [33], gastritis and peptide ulcers [34,35] cardiovascular diseases [36], Crohn’s disease [37], irritable bowel syndrome [38], and gastro-intestinal cancers [21]. Undeniably, today the gut microbiota can be considered as an extra-genomic functional unit that imparts mechanistic control, determining the metabotypes, over the host nutritional health (Figure 2). Besides, there is today a strong need to successfully apply personalized healthcare solutions in deciphering the role of these interactions. 

Indeed, the symbiotic relationships among animals and their gut had been extensively studied by Martin *et al*. who describe at first a top-down view model of the effects of different gut microbiome on murine metabolic profiles [39]. The authors reported that inoculation of germ-free mice with a simplified model of human baby microbiota modifies the physiology of the murine host towards pre-pathological conditions. The metabonomic investigations brought evidence of a functional relationship between the modulation of the bile acid pool, impaired lipid metabolism and gut microbial composition. In particular, the human-derived bacteria were non-adapted to the murine organism and unable to hydrolyze tauro-conjugated bile acids. These metabolic alterations resulted in decreased capability of dietary lipid emulsification and higher intestinal lipid absorption, which in turn altered the proper recirculation and distribution of fat within the organism.

The breadth and the depth of gut microbiome modulations of host biochemistry in this mouse model were further explored by modulating the gut functional ecology with pro-, pre-, and synbiotics [40,41,42]. Gut microbes perform multiple digestive and metabolic functions for the host and these studies revealed that major mammalian metabolic processes are under symbiotic homeostatic control. For instance, the effects of consuming live microbial supplements, probiotics, on the microbial ecology host health and nutritional status have been in the past years investigated [43]. These factors were well elucidated by measuring the transgenomic metabolic effects of exposure to *Lactobacillus paracasei* and *Lactobacillus rhamnosus* probiotics in mice inoculated with a simplified model of human gut microbiota. Changes in dietary carbohydrate and protein processing by gut bacteria with subsequent influence on host lipids and energy metabolism were revealed by the analysis of systemic fluids, liver, stools, and intestinal contents.

As an alternative, the combined use of prebiotics and probiotics may offer superior effects in health maintenance through modulating the microbial functional ecology [42]. Here, metabonomics was able to captured metabolic changes in selected biological compartments, biofluids and liver, which were correlated with modulation in microbial populations. In turn, these microbial effects were associated with changes in various host metabolic pathways including gluconeogenesis, amino acids, methylamine and lipid metabolism. 

Additionally, in a follow up paper, the authors analyzed the effects of single prebiotics and probiotics and their synbiotic effects on the metabolic status of germfree mice during the establishment of a simplified model of human microbiota. In this paper, to properly assess the dietary modulations of the gut microbota at a system level, multicompartmental modeling approach with metabolic imprints from 10 tissue/fluid samples was applied [40]. Interestingly, the authors revealed that the induced microbial changes influenced host lipid, carbohydrate, and amino acid metabolism in every major mammalian biological analyzed compartment. Specifically, it was found that galactosyl-oligosaccharides strongly reduced lipogenesis, triacylglycerol incorporation into lipoproteins and triglyceride concentration in the liver and the kidney. Prebiotic modulation of the gut microbiota also altered transmethylation metabolic pathways in the liver and in the pancreas, with inferred effects on the control of glucose metabolism and insulin sensitivity. In addition, to the previously characterized metabolic effect of probiotics in reducing hepatic glycogen and glutamine levels, these studies brought compelling evidence of decreased adrenal ascorbate levels, with plausible implications in energy homeostasis, antioxidation, and steroidogenesis. 

While deciphering the interactions between the gut microbiota and the host remains a very challenging process, research advances in recent years have positively elucidated key metabolic processes disclosing in depth how the gut microbiota exert control over the host’s biochemistry. Imperative for the development of nutritional management programs and for the amelioration of individual health will be in the very next future to comprehensively embrace the full dynamics of these interactions.

## 3. Conclusions

As previously expressed one of the greatest challenges in modern nutrition research is to decipher how changes in the environment and lifestyle conditions regulate not only human physiology but additionally human ability to attain different nutritional needs. With these aims, the analysis of biofluids by metabonomic means permits the assessment of spatiotemporal interorgan metabolic cross-talk, while the analysis of such information by complex data mining techniques allow the assessment of the functional relationships among different biological compartments. Specifically, the combination of metabolic profiling and multivariate analysis was successfully proved to infer inter-compartment metabolite relationships from plasma, liver, pancreas, adrenal gland and kidney cortex samples [44]. Such a combination of chemometric techniques could provide new research avenues to assess the efficacy of nutritional interventions on targeted organs from the single analysis of a systemic fluid such as the plasma.

Modern nutritional research focuses on health promotion and disease prevention through diets. Nutritional solutions have indeed been developed for the management of several chronic conditions, such as obesity and type 2 diabetes. It is therefore expected that, for this scope, nutrition and health will commonly aim in the very next future into the optimization of food products specifically tailored to match consumer specific needs to promote health and wellness. Metabonomics will provide the required instruments to monitor the metabolic health of consumers and maintaining homeostatic balance. Indeed, adjusting the diet according to the health status will be one of the projected benefits. 

The change of adapting personalized nutritional programs will not only lie in the diagnostic tools, consumer demands and awareness, or measuring technology, but will mostly rely on sound scientific bases. Such processes had been already proven to be well initiated.
